# Supplementary material for: Myristica fragrans Extract Inhibits Platelet Desialylation and Activation to Ameliorate Sepsis-Associated Thrombocytopenia in a Murine CLP-Induced Sepsis Model
Source: Int J Mol Sci. 2023 May 16;24(10):8863. doi: 10.3390/ijms24108863 (PMC10218882; doi:10.3390/ijms24108863)
Supplement: Supplementary file 1 [file ijms-24-08863-s001.zip › ijms-2367028-supplementary.pdf]

Supplementary Figure S1.

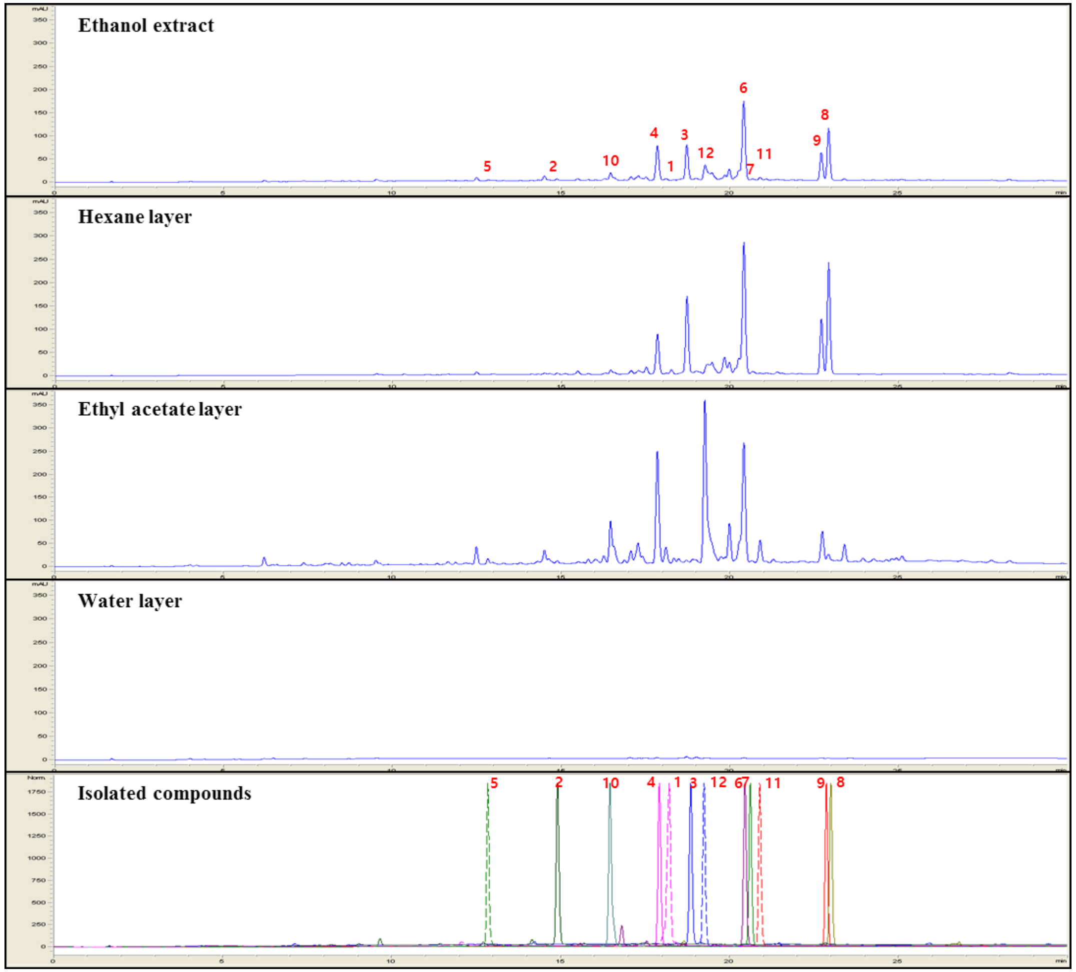

Figure S1. HPLC of extract, fraction layers, and isolated compounds from *M. fragrans.*
Reprinted from Bioorganic & Medicinal Chemistry Letters, Vol. 27, Park et al., Sialidase inhibitory activity of diarylnonanoid and neolignan compounds extracted from the seeds of Myristica fragrans, 3060-3064, Jul 15, 2017, with permission from Elsevier.

Table S1. Hematological analysis of experimental group.

Parameter (Units)		Sham	CLP	CLP+OS	CLP+MF
Leukocytes					
WBC	($\times 10^3$/μl)	5.8 ± 1.1	1.4 ± 0.2	1.8 ± 0.6	1.7 ± 0.5
NE	($\times 10^3$/μl)	0.4 ± 0.3	0.4 ± 0.2	0.6 ± 0.2	0.6 ± 0.2
LY	($\times 10^3$/μl)	5.1 ± 1.0	0.9 ± 0.2	1.0 ± 0.5	1.1 ± 0.5
MO	($\times 10^3$/μl)	0.3 ± 0.1	0.1 ± 0.0	0.1 ± 0.0	0.0 ± 0.0
EO	($\times 10^3$/μl)	0.0 ± 0.0	0.0 ± 0.0	0.0 ± 0.0	0.0 ± 0.0
BA	($\times 10^3$/μl)	0.0 ± 0.0	0.0 ± 0.0	0.0 ± 0.0	0.0 ± 0.0
Erythrocytes					
RBC	(M/μl)	8.9 ± 1.0	9.2 ± 0.1	9.4 ± 0.6	9.1 ± 0.2
Hb	(g/μl)	12.2 ± 1.4	12.5 ± 0.4	13.3 ± 1.3	12.9 ± 0.3
HCT	(%)	45.9 ± 5.3	46.7 ± 1.4	47.3 ± 3.5	46.6 ± 1.5
MCV	(fL)	51.7 ± 1.0	50.6 ± 1.6	44.6 ± 13.8	51.5 ± 2.2
MCH	(pg)	13.8 ± 0.3	13.5 ± 0.3	14.2 ± 0.6	14.2 ± 0.4
MCHC	(g/dl)	26.7 ± 0.7	26.8 ± 0.7	28.2 ± 0.8	27.7 ± 1.3
RDW	(%)	16.0 ± 0.4	16.2 ± 0.5	16.2 ± 0.6	16.3 ± 0.6
Thrombocytes					
PLT	($\times 10^3$/μl)	576.2 ± 16.5	291.0 ± 38.7	389.2 ± 43.5	378.4 ± 44.3
MPV	(fL)	4.2 ± 0.2	4.2 ± 0.1	4.4 ± 0.1	4.4 ± 0.5

Table S2. Mouse primers used for qRT-PCR analysis.

Species	Genes		Primer sequences (5′ to 3′)
Mouse	TPO	Forward	CACAGCTGTCCCAAGCAGTA
		Reverse	CATTCACAGGTCCGTGTGTC
	Cyclophilin A	Forward	GCCGATGACGAGCCCTTG
		Reverse	TGCCGCCAGTGCCATTATG
